# Evaluation of a Telephone-based Physical Activity Promotion Program for Disadvantaged Older Adults

**DOI:** 10.5888/pcd9.110071

**Published:** 2012-02-23

**Authors:** Kristen Hammerback, Glen Felias-Christensen, Elizabeth A. Phelan

**Affiliations:** Department of Health Services, School of Public Health, University of Washington; Department of Health Services, School of Public Health, University of Washington, Seattle, Washington; Department of Health Services, School of Public Health, and Department of Medicine, Division of Gerontology and Geriatric Medicine, University of Washington, Seattle, Washington

## Abstract

**Background:**

Lack of adequate physical activity among older adults has been widely documented. Although interventions aimed at increasing physical activity that are based on behavioral strategies and theories have been shown to increase activity levels among older adults, little is known about responses to these interventions in different population segments.

**Community Context:**

The Physical Activity for a Lifetime of Success (PALS) program attempted to translate a telephone-based, motivational support program for physical activity, Active Choices, for use by a low-income, ethnically diverse population of older adults living in southeast Seattle. This article describes the evaluation of PALS at the end of the 5-year program.

**Methods:**

Evaluation data included a data set of participant physical activity assessments; internal study documents; and interviews with key PALS stakeholders, participants, volunteers, and people eligible for PALS who declined to enroll when invited.

**Outcome:**

PALS demonstrated improved physical activity levels among the sedentary older adults who participated in the program, but the PALS model did not appeal widely to a diverse, low-income target population. Extensive recruitment efforts resulted in a low number of participants, and attempts to recruit peer volunteers were largely unsuccessful.

**Interpretation:**

Considering the resources required to engage both participants and volunteers, PALS does not appear to be a sustainable model for delivering support for physical activity to community-dwelling minority and low-income older adults.

## Background

Despite evidence demonstrating the benefits of physical activity for older adults, including the prevention and control of chronic diseases (1,2) and reduction in risk of falls (3-5), most older adults do not achieve recommended levels of physical activity (6). Identifying ways to increase physical activity in this large and growing segment of the population is a public health priority.

Physical activity interventions based on behavioral strategies and theories increase levels of participation among older adults (7). However, little is known about how responses to these interventions differ depending on the population segment involved, because few have been disseminated in nonresearch settings.

The Physical Activity for a Lifetime of Success (PALS) program was based on Active Choices, an evidence-based program developed at Stanford University. PALS aimed to increase the physical activity levels of sedentary older adults through one-on-one telephone support delivered by adult volunteers who were trained in motivational interviewing. Participants received twice-monthly, 20- to 30-minute telephone calls for the initial 6 months, followed by monthly calls for up to 6 additional months. Volunteers did not receive any stipend or nonmonetary compensation. A description of the program and the first phase of its implementation can be found elsewhere (8).

The objective of this study was to understand the appropriateness of the PALS intervention for the target audience (an older, low-income, ethnically diverse population), whether it was successful in improving Participants' levels of physical activity, barriers and motivators for participation, and lessons learned for implementing similar programs.

## Community Context

PALS was developed and implemented between March 2005 and September 2009 through a partnership between the University of Washington Health Promotion Research Center (HPRC) and Senior Services; funding was provided through a cooperative agreement with the Centers for Disease Control and Prevention (CDC).

### Recruitment challenges

Recruiting was a challenge during all 3 phases of the 5-year study (8). In the initial phase, beginning March 2005, we used a randomized controlled trial design to test PALS and recruited participants via "green prescription," a written recommendation from the physician for increased physical activity (9). During this phase we recruited people aged 65 years or older who had diabetes; who were living in an ethnically diverse, low-income neighborhood of Seattle, Washington; and who were receiving care at 1 of 2 community clinics. We recruited participants during their usual visits with their primary care provider. Participants were randomized to receive either an immediate or delayed intervention that consisted of a physical activity assessment, brief counseling by a primary care provider, and a referral to the PALS program. Recruiting participants using this strategy was unexpectedly difficult. Although the initial expectation was that half or more of the patients approached by their doctors would agree to participate, only 14 people (21%) enrolled, despite encouragement from primary care providers and follow-up calls from research staff.

During the second phase, beginning in 2006, we modified recruitment criteria to include younger adults (aged ≥50 y) and people who did not have diabetes, and we changed the research design to a pre-post comparison. Recruitment efforts targeted the 900-plus members of the Southeast Seattle Senior Center (SSSC), a neighborhood center that provides programs for community-dwelling older adults. SSSC, which serves a largely low-income, ethnically diverse population, was the operations hub for the PALS program. Letters and PALS brochures were mailed to 150 randomly selected people on the senior center's mailing list, and telephone calls were made to each recipient within a week after the mailing. Other recruitment methods included asking a health educator from recruitment phase 1 community clinics to recommend program enrollment to eligible patients, publishing articles in neighborhood newspapers, distributing brochures at senior housing sites, and speaking at community events. Although enrollment was lower than expected (48 enrolled by January 1, 2008), results demonstrated the effectiveness of PALS in increasing physical activity levels among participants, many of whom expressed strong enthusiasm for the program. A final phase, aimed at reaching as many eligible adults as possible, began in spring 2008.

Phase 3 outreach efforts centered on recruiting via community organizations that served older adults throughout King County, Washington, including Asian Counseling and Referral Services (serving primarily Asian populations), SeaMar Community Health Centers (serving a largely Latino/Latina population), the US Veterans' Administration MOVE program, the King County Employee Wellness Initiative, and Visiting Nurse Services of the Northwest. PALS project staff also presented information sessions about the program at locations visited by older adults or locations where they resided, such as local civic clubs, assisted living and retirement communities, and churches and synagogues. Audience sizes for these outreach efforts ranged from a few to 50 people.

### Evaluation objectives

We designed the PALS evaluation to assess the extent to which PALS met 2 key objectives: to increase the physical activity level of the program's participants and to successfully adapt an evidence-based model (Active Choices) for a diverse, low-income, older adult population. Secondary evaluation aims included determining what factors may have interfered with these objectives being fully met, and considering lessons that may inform future attempts to implement heath promotion programs among diverse, low-income, older adult populations.

An external evaluator designed the PALS evaluation in consultation with the research team. All data collection instruments and procedures were approved by the University of Washington institutional review board.

## Methods

### Data sources

**Physical activity outcome measures database**

Participants' physical activity level was measured by using the Rapid Assessment of Physical Activity (RAPA) questionnaire (10) (available on request from corresponding author). The RAPA measures frequency and intensity of physical activity in the areas of aerobic endurance, strength, and flexibility. A RAPA score of 1 to 3 corresponds to minimal physical activity, a score of 4 or 5 is associated with some health benefit, and a score of 6 or 7 meets the CDC standard for physical activity. The RAPA instrument was designed for use in clinic settings and has been validated (10).

**Program process documents**

Formal and informal proposal-stage and preparatory reviews and assessments, progress reports, and implementation-related e-mails, reviews, and assessments were used as data sources. Examples include the health-planning demographic data used to assess the community's need for a physical activity program, periodic progress reports prepared for the funder, and archived e-mails between the PALS coordinator and volunteers.

**Interviews**

We conducted structured, one-on-one interviews with 25 key PALS stakeholders, including past and present members of the research team, the current PALS coordinator, community partners, PALS volunteers (telephone "buddies"), and PALS participants (interview questions available on request from corresponding author). We also interviewed 10 program "nonjoiners," defined as people recruited for PALS who chose not to enroll.

### Data collection

To measure the effectiveness of PALS for increasing Participants' physical activity levels, RAPA assessments were completed on enrollment and at 3, 6, and 12 months postenrollment. The initial RAPA assessment was conducted in person; follow-up RAPA assessments were administered by telephone. Previous work assessing the validity and reliability of in-person and telephone-administered RAPA assessments supported these approaches (11). All RAPA scores were collected by the SSSC PALS coordinator.

To review the large collection of PALS-related documents, 2 PALS evaluation team members read through and summarized nonconfidential electronic and paper files belonging to PALS program coordinators and researchers. Evaluators conferred frequently to ensure consistency of interpretation.

To capture the views and experiences of PALS stakeholders, an interview guide was constructed and pretested. Stakeholders were asked to describe their experience with PALS, including challenges encountered and lessons learned. Each semistructured interview, completed by 1 of 2 evaluators, lasted between 45 and 60 minutes. Conversations were not audiotaped, but interviewers prepared a written summary immediately after the interview.

As part of a separate study on motivations and barriers to older adults' participation in physical activity programs, we conducted semistructured interviews with 2 sets of subjects: participants in the PALS program ("joiners") and people who expressly declined participation ("nonjoiners"). For this evaluation, we analyzed only those parts of the interviews related to the PALS experience. We asked interviewees what brought them to PALS, what aspects were most appealing, and what suggestions they had for improvement. Nonjoiners answered questions about barriers to joining and aspects of PALS they found problematic. All interviews were conducted in person, usually in the participant's home. Interviews were audiotaped and professionally transcribed. We conducted 20 interviews (10 each with joiners and nonjoiners.)

We contacted PALS volunteers via regular mail or e-mail and asked them to complete a brief written questionnaire about their PALS experience. The questionnaire addressed motivations for volunteering to be a telephone buddy, experiences with making telephone calls and interacting with participants, and reasons for ending involvement. We had current contact information for 15 of the 39 PALS volunteers. Of these 15, 8 completed a survey.

### Data analysis

To measure the association of PALS participation with increased levels of physical activity, we compared baseline and 6-month RAPA assessment scores. The 6-month interval was chosen because PALS was initially designed as a 6-month program with the option to continue up to 6 additional months. Therefore, many participants completed the program before being administered the 12-month RAPA assessment. Analysis followed a pre-post design, using a McNemar test for matched pairs to assess differences between RAPA scores at enrollment and follow-up. Data were analyzed using SPSS version 15 (SPSS, Inc, Chicago, Illinois).

Qualitative data were analyzed by using a grounded theory approach. Two evaluation team members read every interview summary, interview transcript, and internal document, and met frequently to discuss and verify agreement on themes, particularly those present in more than 1 source. To ensure accuracy and consensus, we compiled a list of key themes and sent it to the remaining members of the evaluation team for review.

## Outcomes

### Effect of PALS on physical activity levels

Although 131 people enrolled in PALS and received a baseline RAPA score, only 89 (68%) were also administered the RAPA assessment at 6 months. The remaining 42 participants either dropped out before 6 months (n = 20) or were unable to be reached to participate in a 6-month RAPA assessment (n = 22). Of these 89 participants, 12 (13%) had baseline scores of 6 or 7, which indicated that they met the CDC standard for physical activity. At 6-month follow-up, 22 people (25%) met the CDC physical activity standard, a significant increase (McNemar χ^2^ = 4.545, *P* = .05) (Table 1). The 3 types of physical activity measured showed similar increases.

### Translating Active Choices into PALS

Review of program process documents indicated that, when initially considering Active Choices as a program model, HPRC researchers did not conduct formal target audience research to assess whether a telephone-based, volunteer, peer support model would be suitable or appealing to low-income, ethnically diverse, older adults living in southeast Seattle. Instead, researchers consulted members of the HPRC Community Advisory Board who represented PALS target communities. These members endorsed the choice but did not formally test it among potential participants.

Post-PALS interviews with members of the community indicated that many felt the PALS model did not meet their wants or needs. For example, some Latino older adults reported that members of their community would not feel comfortable talking regularly on the telephone to someone with whom they did not also have a face-to-face relationship. Some older adults who declined an invitation to join PALS voiced concern about talking openly with someone much younger or of a different race and doubted that someone so different could really understand the challenges they faced in trying to start a regular routine of physical activity. Finally, many did not like the idea of receiving telephone calls from strangers.

Interviews with 10 former PALS participants also indicated a lack of enthusiasm for a telephone-based model, and most of the nonjoiners indicated that communicating only via telephone was a major reason for declining to enroll.

### Recruitment

**PALS participants**

During the first year of PALS, 2005, 14 of the 65 older adults invited to enroll did so (Figure). In years 2 and 3, invitations to 150 members of the SSSC yielded 4 new participants; continued referrals from the 2 clinics and word of mouth resulted in an additional 30 enrolled participants. In the summer of 2008, 2 members of the PALS research team gave 99 presentations and talks at locations visited by older adults or locations where they resided. As a result of this intense 3-month period of recruitment, 29 people enrolled in PALS, about 1 person for every 3 presentations. Furthermore, we produced and disseminated a short video with information about the program and testimonials from satisfied participants, and distributed articles and profiles highlighting PALS to local newspapers and newsletters throughout 2008 and 2009. These efforts ultimately added 54 participants by September 2009, bringing the total enrolled to 131.

**Figure.**
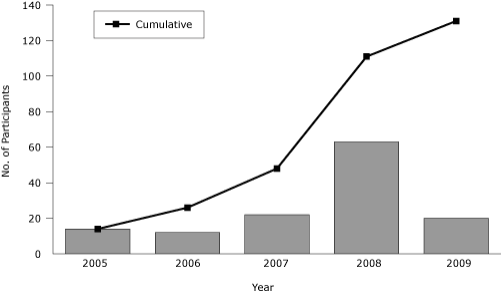
Enrollment in Physical Activity for a Lifetime of Success (PALS) program, per year and cumulative, 2005-2009. Data from PALS participant database.

**Table:** 

Year	Participants Per Year	Cumulative No. of Participants
2005	14	14
2006	12	26
2007	22	48
2008	63	111
2009	20	131

**Volunteers**

Interviews with PALS stakeholders indicated that attracting and keeping reliable, enthusiastic volunteers was challenging and time-consuming. Originally, PALS sought volunteers who could act as peer mentors, ideally older adults with regular physical activity routines, preferably living in the same community and with similar backgrounds to those of participants, who would presumably enjoy the process of encouraging another elder to become more active. As a result of the dearth of potential volunteers meeting these criteria, we adopted broader criteria, which yielded a volunteer pool that was younger, less diverse, and more educated than the average PALS participant (Table 2).

The 8 volunteers we interviewed indicated that developing rapport with participants was an ongoing challenge, as was contacting participants ("My participants never returned my calls!"). Most of these volunteers felt discouraged at what they perceived to be the difficulty of making a strong connection with their assigned participants, and few described the experience as satisfying ("It was a lot of work without much return.").

### Implementation

Both interviews and process documents suggested that PALS implementation was hampered by high turnover among research team and program staff, because the turnover threatened the program's institutional memory and stability. Furthermore, the PALS coordinator felt overwhelmed by the challenges of collecting and recording data from volunteers. Although volunteers were asked to commit to being a telephone buddy for a minimum of 1 year, many ended their involvement within a few months of completing the PALS telephone buddy training, requiring the coordinator to hold many more trainings and do more outreach than originally anticipated.

## Interpretation

Although PALS was effective in increasing physical activity levels among participants, recruitment of participants and volunteers required too much time, effort, and funding for sustainability. Therefore, the program was discontinued at the end of its research cycle in September 2009.

### Possible reasons for difficulty in recruiting participants

Two issues may underlie the challenges we encountered in recruiting participants. First, few people in our target communities may have been in the appropriate stage of behavior change, meaning that the available pool from which to recruit was limited. We did not assess stage of change as part of PALS recruitment; the research team decided that making this assessment would reduce the practical, real-world translational effort and make this project similar to a research study. Although a stage-of-change assessment may have helped us direct our recruitment efforts more strategically (12), making this type of assessment was not feasible in the context of our recruitment approach during phase 3. However, organizations planning to implement such programs may find that assessing readiness for change presents an opportunity to help both program operators and potential participants understand the person-program fit.

Second, the PALS model was not appealing to the study's target population. Community partner stakeholder interviews identified barriers, including the lack of face-to-face interaction between participants and their volunteer telephone buddies and the frequent differences between these 2 groups in their cultural identities, ages, and socioeconomic levels. Incorporating more input from members of the target population early on in the choice of model may have indicated that these factors would be barriers (13). PALS may have been more successful if we had performed a preliminary needs assessment of our target audience with older adult members of our partner organizations to inform the selection of an appropriate program model. In doing so, we may have recognized that a telephone-based intervention was not appropriate for a sizeable proportion of our intended audience.

### Possible reasons for difficulty in recruiting and retaining volunteers

PALS experienced challenges typical for nonprofits in recruiting and retaining volunteers (14), and volunteers tended to be younger, more educated, and less racially diverse than PALS participants. Previous studies indicate that volunteers in health promotion programs are often younger and more highly educated than participants (14,15). Because we did not conduct preliminary market research among possible volunteers, the PALS telephone buddy model may not have appealed to the study's target volunteer population (those who resembled the target population for the PALS program).

The PALS evaluation was limited by the low response rate for our volunteer interviews (21%), which may have limited the range of perspectives reflected. However, the use of an objective external evaluator, triangulation of key findings through multiple data sources, the mix of qualitative and quantitative data elements, and the elicitation of a range of key perspectives were strengths of the PALS evaluation.

## Conclusions

PALS achieved its objective of improving physical activity levels among the sedentary older adults who participated in the program, but the PALS model did not appeal widely to our diverse, low-income target population. Because of the amount of time required to recruit participants and volunteers, we do not consider PALS a sustainable model for delivering support for physical activity to community-dwelling, minority, and low-income older adults. Developing ways to reach this target audience, however, is worthy of continued study.

## Figures and Tables

**Table 1:** Distribution of Rapid Assessment of Physical Activity (RAPA) Scores for PALS Participants (N = 89) at Enrollment and at 6 Months Following Enrollment, 2005-2009^a^

RAPA Score^b^	At Enrollment, %	≥6 Months Following Enrollment, %
1-3	62	22
4 or 5	25	48
6 or 7	13	25

a Calculated using McNemar test for matched pairs, *P* = .05 for RAPA scores dichotomized to sufficiently active (yes/no).

b RAPA scores of 1-3 correspond to minimal physical activity, 4 or 5 are suboptimal with some benefit, and 6 or 7 are optimal (based on Centers for Disease Control and Prevention standards for physical activity).

**Table 2:** Descriptive Characteristics of Physical Activity for a Lifetime of Success (PALS) Participants and Volunteers

Characteristic	Participants^a^ (n = 55)	Volunteers (n = 39)
Mean age, y	70	55
% White	51	76
% College graduate	29	81

a Only participants for whom demographic data were collected were included in this analysis. Demographic data were regularly collected beginning August 2008.
